# Persistence of the efficacy of copper oxide wire particles against *Haemonchus contortus* in grazing South African goats

**DOI:** 10.1016/j.vetpar.2012.06.018

**Published:** 2012-11-23

**Authors:** A.F. Vatta, P.J. Waller, J.B. Githiori, G.F. Medley

**Affiliations:** a Onderstepoort Veterinary Institute, Private Bag X05, Onderstepoort 0110, South Africa; b National Veterinary Institute, Department of Parasitology (SWEPAR), Uppsala SE-751 89, Sweden; c International Livestock Research Institute, P.O. Box 30109, Nairobi 00100, Kenya; d University of Warwick, School of Life Sciences, Coventry CV4 7AL, United Kingdom

**Keywords:** Anthelmintic resistance, Copper oxide wire particles (COWP), Goats, *Haemonchus contortus*, Persistent efficacy

## Abstract

A study was conducted to examine the duration of anthelmintic effect of copper oxide wire particles (COWP) in grazing goats, as data for the persistence of efficacy of COWP in this host species is limited. Forty-eight indigenous male goats were infected naturally by grazing them on *Haemonchus contortus*-infected pasture. When the faecal egg count (FEC) in the goats was 3179 ± 540 eggs per gram of faeces (mean ± standard error), half the animals were treated with 4 g COWP (day 0; mean live weight = 25.5 ± 0.8 kg). Eight treated (COWP) and eight non-treated (CONTROL) goats were removed from the pasture on each of days 7, 28 and 56, maintained for 27 or 29 days in concrete pens and then humanely slaughtered for nematode recovery. Mean liver copper levels were in the high range in the goats removed from pasture at day 7 (treated: 191 ± 19.7 ppm; untreated: 120 ± 19.7 ppm; *P* = 0.022), but had dropped to normal levels at days 28 and 56. The mean *H. contortus* burdens of the treated versus the non-treated goats were, respectively, 184 ± 48 and 645 ± 152 for the goats removed from pasture at day 7 (71% reduction; *P* = 0.004), 207 ± 42 and 331 ± 156 at day 28 (37% reduction; *P* = 0.945) and 336 ± 89 and 225 ± 53 at day 56 (−49% reduction; *P* = 0.665). Weekly monitoring of FECs after treatment until slaughter indicated that the COWP-treated goats had lower FECs than the controls, the treatment main effect being significant at days 7, 28 and 56 (*P* < 0.01). The day main effect and the treatment × day interaction were only significant for the goats removed from pasture at day 28 (*P* ≤ 0.001). Packed cell volumes increased during the course of the experiment (day, *P* < 0.001), but the treatment main effect was significant only for the goats removed from pasture at day 28 (CONTROL 28 d, 28.65 ± 0.52% < COWP 28 d, 31.31 ± 0.52%; *P* < 0.001). No differences in live weight between groups were considered to be of any practical significance. The study indicated that persistence of efficacy of COWP is limited in goats, extending at most to 28 days after treatment. However, repeated COWP administration at three-month intervals may be safe, given that liver copper levels return to normal two to three months after COWP treatment.

## 1 Introduction

Disease caused by *Haemonchus contortus* is one of the major constraints to the production of sheep and goats in the tropics and subtropics, and causes substantial losses to farmers worldwide. The anthelmintic properties of copper-containing compounds have been known for a long time (Wright and Bozicevich, 1931), but the worldwide increase in anthelmintic resistance has prompted more recent investigations into the renewed use of copper as an anthelmintic (Burke et al., 2007). Specifically, investigations have focused on copper oxide wire particles (COWP) which have been shown to have an anthelmintic effect against abomasal nematodes, particularly *H. contortus* (Bang et al., 1990). They represent a potentially cheap alternative to anthelmintics for small-scale farmers in the developing world, if the use of COWP can be successfully integrated into worm control programmes.

Only one study (Galindo-Barboza et al., 2011) has specifically examined the persistence of efficacy of COWP based on worm counts in sheep. Recent data from goats managed under communal farming conditions suggest that egg counts are reduced two weeks, but not six weeks, after treatment with COWP (Spickett et al., 2012). However, no worm count data are available on the duration of efficacy of COWP in groups of goats subjected to similar levels of parasite exposure, nutrition and management. The present study therefore sought to examine the effect of COWP treatment in goats treated and removed from infective pasture at three different stages, namely at 7, 28 and 56 days post treatment.

## 2 Materials and methods

The use of animals for this experiment met the requirements of the Onderstepoort Veterinary Institute Animal Ethics Committee.

### 2.1 Preparation of infected pasture

A 0.67 ha pasture of star grass (*Cynodon incompletus* Nees) at Onderstepoort Veterinary Institute, Pretoria was utilized for the study in 2006–2007. In the spring of 2006, six months prior to the start of the actual experiment, the grass was cut and fertilized. The pasture was irrigated through the spring and summer until the conclusion of the experiment in the following autumn if less than 25 mm rain fell during the previous week. Rainfall data were collected at Onderstepoort Veterinary Institute while temperature data were obtained from the South African Weather Service for central Pretoria, which is approximately 16 km south of the Institute.

Since the pasture had not been used for animal grazing for several years prior to the experiment, it was seeded with *H. contortus* larvae by grazing infected sheep on it. Initially, twenty indigenous sheep were purchased from a commercial vendor, transported to Onderstepoort Veterinary Institute and maintained in concrete pens which were swept clean daily to preclude accidental nematode infection. The animals were fed a commercial pelleted feed and lucerne (*Medicago sativa*) hay and the animals had free access to water. The sheep were dewormed with 10 mg/kg fenbendazole (Panacur BS^®^, Intervet South Africa) and 7.5 mg/kg levamisole (Tramisol^®^, Coopers, Afrivet Business Management, South Africa) daily for 5 days, followed by 0.3–0.5 mg/kg ivermectin (Ivomec Injection^®^, Merial South Africa) administered 6 days and 13 days after the combination treatment with fenbendazole and levamisole. Thirty-three days later, the animals were infected with 5000 third-stage larvae of a susceptible strain of *H. contortus* given as 1000 larvae per day for five days, as low-level, trickle dosing has been shown to be the optimal method for achieving establishment of parasites (Barger et al., 1985; Dobson et al., 1990). When the infections were patent in the late spring period (on day −82 relative to the start of the experiment), the sheep were transferred to the pasture where they were grazed from Monday to Friday from 8.00 am to 3.00 pm. For security reasons, the sheep were maintained in their pens overnight and on the weekends, where they received hay and pellets and free access to water.

### 2.2 Experimental goats

Forty-eight indigenous intact (*n* = 20) and castrated (*n* = 28) male goats were purchased from an experimental farm near Pietermaritzburg, South Africa, the same farm from which resistance to anthelmintics had been reported in Van Wyk et al. (1989) and Vatta et al. (2009), and transported to Onderstepoort Veterinary Institute where they were maintained and fed in a similar manner to the sheep. The goats were dewormed with a combination of 7.5 mg/kg levamisole and 7.5 mg/kg rafoxanide (Nem-a-rid^®^, Intervet South Africa) on day −114 relative to the start of the experiment. When faecal egg counts (FECs) were carried out on the goats 9 days after treatment, the reduction in egg count was 40% (Table 1). Third-stage larvae recovered following culture of the faeces were identified using the key of Van Wyk et al. (2004) and proportionally belonged to the following genera: 61% *Haemonchus*, 24% *Teladorsagia*/*Trichostrongylus* and 15% *Oesophagostomum* (*n* = 100). The goats were treated with 0.4 mg/kg moxidectin (Cydectin Injectable^®^, Fort Dodge Animal Health) 5 days later and the FECs were reduced by 85% when determined 14 days after this treatment. Only two larvae were recovered on faecal culture following the second treatment and both were *Haemonchus* spp.

The goats were maintained in pens until day −51 when they were moved to the pasture seeded with *H. contortus* larvae by the sheep. The goats were grazed together with the sheep until day −2 of the experiment, when the sheep were removed from the pasture. The FECs of the goats were checked weekly until day −2 when their mean FEC was 3179 ± 540 epg. Two days later, on day 0 (28 February 2007), the 48 goats were allocated to six experimental groups for treatment/non-treatment and date of removal from pasture. The goats were paired for average live weight and FECs for the two sampling dates (days −9 and −2) preceding the date of treatment. Eight clusters were formed consisting of three pairs of goats with similar live weight and FEC. A pair of goats was randomly selected from a cluster and allocated to one of three dates of removal from pasture (7 d, 28 d or 56 d), one goat was allocated to treatment (COWP) and the other to non-treatment (CONTROL). This process was repeated for the remaining pairs within a cluster so that a goat from each cluster was ultimately allocated to each of the six experimental groups, and repeated for the remaining seven clusters.

Treatment was with 4 g COWP (Copinox Ewe/Calf^®^, Animax Ltd., UK). The mean live weight of the goats two days prior to treatment was 25.5 ± 0.8 kg and the animals were between 8 and 11 months old. Sets of eight treated and eight untreated goats were removed from the pasture on each of days 7, 28 and 56. Following removal from pasture, the goats were maintained for 27 or 29 days in concrete pens that precluded further nematode infection and were then humanely slaughtered for worm recovery and testing for tissue copper levels.

### 2.3 Experimental monitoring and parasitological procedures

Five days after treatment and on a weekly basis after that, the animals were weighed using a Ruddweigh 500 Portable Weighscale (Ruddweigh International Scale Co., Australia) and scored for body condition score on a scale of 1 (thin) to 5 (fat) (Russell, 1984; Williams, 1990). Faecal samples were collected from the rectum for FEC using a modified McMaster method (Reinecke, 1983) and blood samples were collected for packed cell volume (PCV) determination by the microhaematocrit method (Vatta et al., 2007).

Worms were recovered at slaughter from the abomasum and small intestine of each goat according to the methods of Wood et al. (1995). Two 10% aliquots of the contents of each organ were prepared and the nematodes recovered and counted from these aliquots. The first 15 worms to be counted per aliquot were mounted on microscope slides for identification according to Visser et al. (1987). The mucosae of the abomasum and small intestine were digested using the peptic digestion technique described by the Ministry of Agriculture, Fisheries and Food (1986). All the nematodes in the digested material were recovered and counted while the first 15 nematodes to be counted were identified. The average worm count for the two aliquots of each organ was determined and multiplied by 10. This number was added to the count for the digested material to give the total number of nematodes for that organ.

Samples from the liver, kidney, muscle and faeces were obtained at slaughter and were analysed for copper on a wet matter basis according to the method of Boyazoglu et al. (1972). This comprised the use of an acid digestion technique and the values were determined on an atomic absorption spectrophotometer (GBC 908 AA, GBC Scientific Equipment, Dandenong, Australia).

### 2.4 Statistical analysis

Using GenStat^®^ (Payne et al., 2011a), restricted maximum likelihood (REML) repeated measurement analysis (Payne et al., 2011b) was applied to the FECs, PCVs, live weights and body condition scores separately for the goats removed from pasture on days 7, 28 and 56 to model the correlation over the duration of the experiment. The fixed effects were specified as day, treatment group and the day × treatment interaction. The random effects were specified as goat and the goat × day interaction. An autoregressive model of order 1 (AR1) to allow for changing variances over days was found to best model the correlation over time. Testing was done at the 1% level of significance as the treatment variances were not homogeneous. Values for day −2 were included as covariates for all variables examined. Castration was included as a factor where significant (*P* < 0.01). Unless otherwise indicated, the adjusted means and standard errors of the means are presented for the PCVs, live weights and body condition scores. The FECs were log_10_ transformed to stabilize the group variances prior to statistical analysis. The adjusted means and standard errors for the untransformed FECs are presented, with statistical inferences based upon the transformed data.

An analysis of variance (ANOVA) was carried out on the log_10_ worm count data and the data for the levels of copper in the organs at slaughter, examining the effects of COWP treatment on these variables. The ANOVA was also applied to the FEC and PCV data to determine whether the values differed for specific days. The effects of castration and day −2 values were examined but found not to be significant. Fisher's protected least significant difference test was used to separate the means at the 1% level. As with the FECs, the untransformed values are reported in the text and tables.

The percentages reduction in worm count in the treated groups relative to the controls were calculated according to the formula, ((*C* − *T*)/*C*) × 100, where *C* and *T* are the untransformed, arithmetic means of the untreated and treated groups, respectively.

## 3 Results

Mean liver copper levels tended to be higher in the treated goats than in the controls removed from pasture 7 days post treatment and slaughtered 28 days later (*P* = 0.022; Table 2), but did not differ in the goats removed from pasture 28 or 56 days after treatment and slaughtered 28 days later. There were no significant differences in the copper levels in the kidney, muscle and faecal samples at slaughter (*P* ≥ 0.09).

**Fig. 1:**
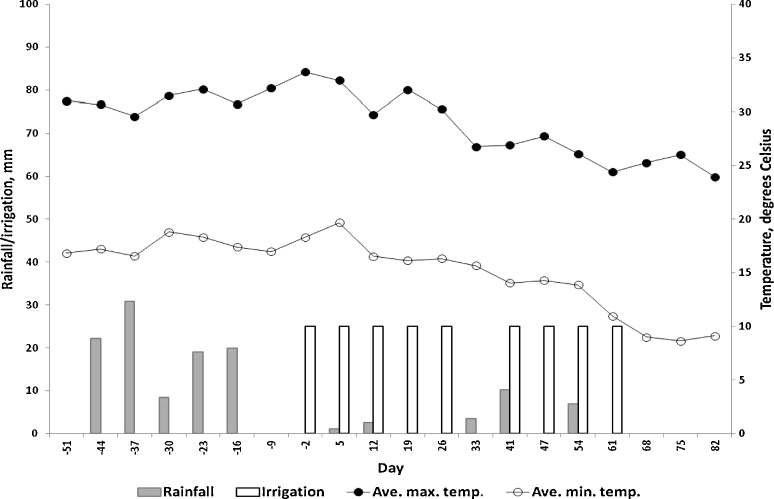
Total weekly rainfall (grey bars) and average weekly minimum (○) and maximum (●) temperatures for Onderstepoort. The pasture on which the goats were grazed was irrigated with 25 mm water on the days indicated (white bars).

The climatic data for the period of the study are presented in Fig. 1. Temperatures declined during the experimental period, as did rainfall. There was a consequent need for artificial irrigation of the pasture.

**Fig. 2:**
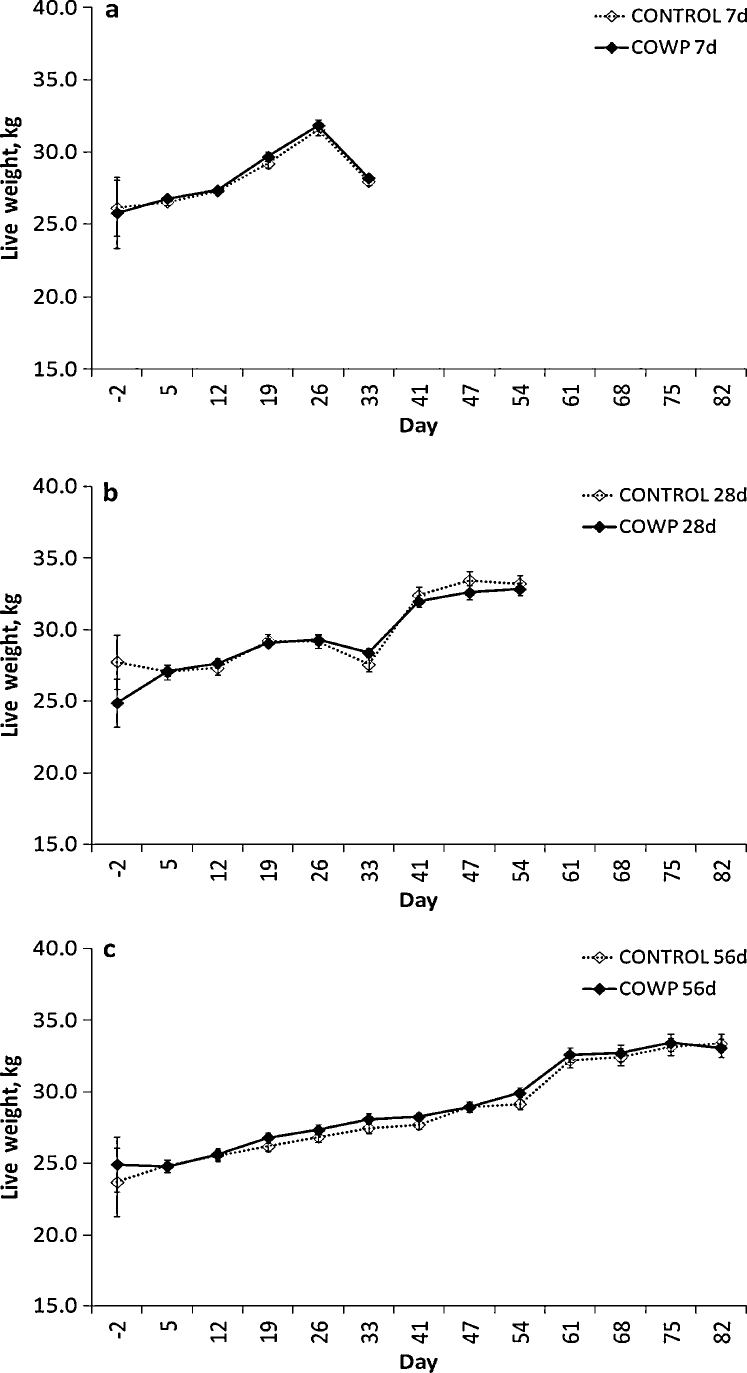
Live weights in kilograms for groups of goats (*n* = 8) treated with 4 g copper oxide wire particles (♦, COWP) on day 0 and removed from *Haemonchus contortus*-seeded pasture on day 7 (a), day 28 (b) or day 56 (c) post treatment and the respective control groups (◊, CONTROL). Adjusted means ± standard errors of the means for day 5 onwards are presented. Day 2 means ± standard errors represent the unadjusted values used as covariates in the analysis.

The effect of castration was not significant for FEC, PCV or body condition score and it was only included as a factor in the analysis for live weight. While the effect of castration was significant for all three sets of goats, the differences were minor (0.35–0.6 kg), were inconsistently different between the groups and were of no practical significance. The goats grew over the course of the experiment (day, *P* < 0.001 for all three sets of goats; Fig. 2), but for reasons that are unknown, the 7 d goats decreased in weight in the week preceding slaughter (on days 34 and 36). The treatment main effect was significant for the groups removed at 28 days and 56 days post treatment (*P* < 0.001) but again these differences were of little biological importance. The interactions of day × treatment and day × castration were not significant for any of the three sets of goats (*P* ≥ 0.028).

The treatment main effect on body condition score was not significant for any of the three sets of goats (*P* > 0.13) and the day main effect was only significant for the goats removed from pasture at 56 days (*P* = 0.002). The latter group increased in body condition score from 3.1 ± 0.1 at the start of the experiment (day −2) to 3.5 ± 0.1 at the end (day 82), whereas the goats removed from pasture at day 7 remained around 3.1 ± 0.05 (*P* = 0.186) and those removed at day 28 around 3.2 ± 0.03 (*P* = 0.066) for the duration of the experiment (data not shown). The interaction of day × treatment was not significant for any of the three sets of goats (*P* > 0.02).

**Fig. 3:**
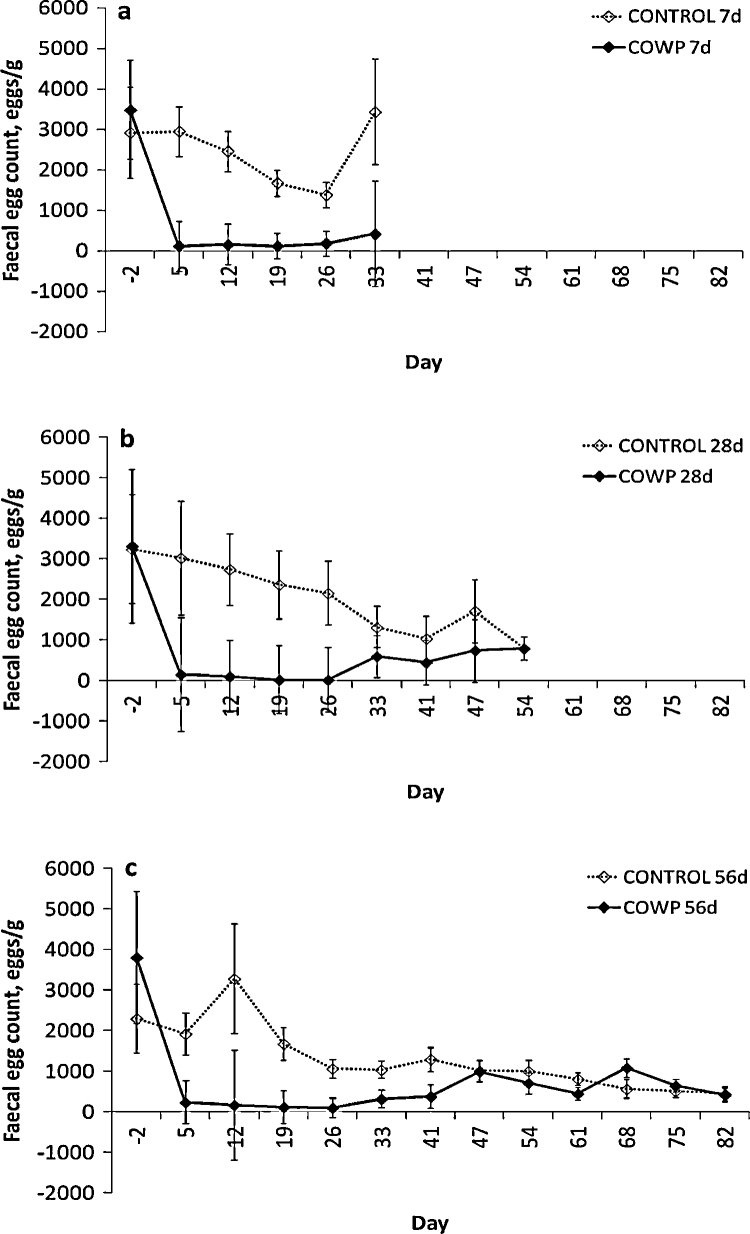
Faecal egg counts in eggs per gram of faeces for groups of goats (*n* = 8) treated with 4 g copper oxide wire particles (♦, COWP) on day 0 and removed from *Haemonchus contortus*-seeded pasture on day 7 (a), day 28 (b) or day 56 (c) post treatment and the respective control groups (◊, CONTROL). Adjusted means ± standard errors of the means for the untransformed data for day 5 onwards are presented in the figure, but statistical inferences in the text are based upon log_10_ transformed data. Day 2 means ± standard errors represent the unadjusted values used as covariates in the analysis.

FECs were reduced in those goats treated with COWP and the treatment main effect on REML analysis was significant for the goats removed from pasture on day 7 (*P* < 0.001; Fig. 3a), day 28 (*P* = 0.003; Fig. 3b) and day 56 (*P* = 0.001; Fig. 3c). Egg counts remained lower in the treated goats until day 26 and started to increase again on day 33. Egg counts declined over the period of the experiment in the control animals and the day main effect tended towards significance for day 7 (*P* = 0.012), was significant for day 28 (*P* = 0.001), but was not significant for day 56 (*P* = 0.070). The day × treatment interaction tended towards significance for day 7 (*P* = 0.019), was significant for day 28 (*P* < 0.001) and was not significant for day 56 (*P* = 0.074). The ANOVA indicated that the FECs for the COWP-treated goats were lower than the controls for the 7 d goats on days 5, 12 and 19 (*P* ≤ 0.004), for the 28 d goats on days 12, 19 and 26 (*P* ≤ 0.005) and for the 56 d goats on days 5, 12, 19 and 26 (*P* ≤ 0.009).

**Fig. 4:**
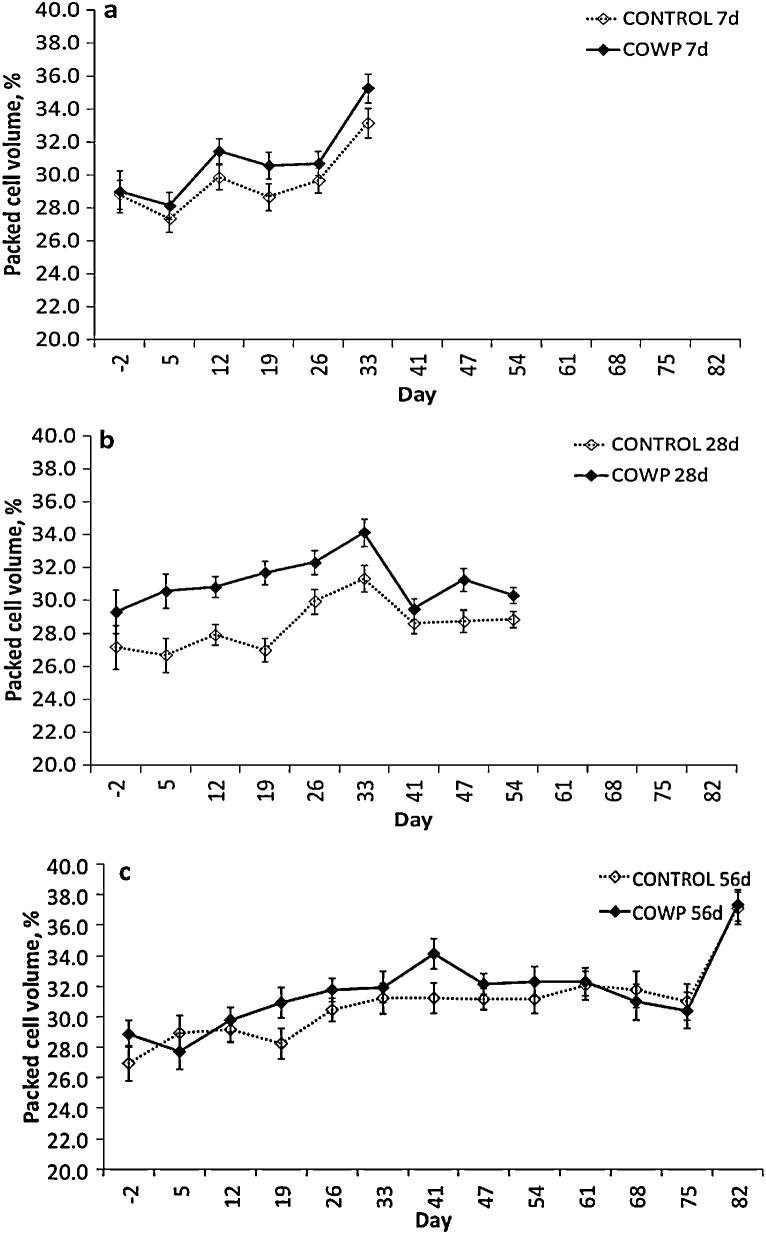
Packed cell volumes as percentages for groups of goats (*n* = 8) treated with 4 g copper oxide wire particles (♦, COWP) on day 0 and removed from *Haemonchus contortus*-seeded pasture on day 7 (a), day 28 (b) or day 56 (c) post treatment and the respective control groups (◊, CONTROL). Adjusted means ± standard errors of the means for day 5 onwards are presented. Day 2 means ± standard errors represent the unadjusted values used as covariates in the analysis.

The PCVs increased during the course of the experiment and, on REML analysis, the day main effect was significant for all three sets of goats (*P* < 0.001; Fig. 4). The treatment main effect was significant for day 28 (CONTROL 28 d, 28.65 ± 0.52% < COWP 28 d, 31.31 ± 0.52%; *P* < 0.001; Fig. 4b), but not for the goats removed from pasture at 7 d and 56 d (*P* > 0.04). The day × treatment interaction was not significant for any of the three sets (*P* > 0.02). On ANOVA, the COWP-treated goats for the groups removed from pasture at day 28 had higher PCVs on days 5, 12, 19 and 47 (*P* ≤ 0.01).

*H. contortus* predominated in the nematodes recovered at slaughter, with an overall mean count of 321 ± 45 worms (Table 3). Six to 23 percent of the *H. contortus* recovered were fourth-stage larvae. Based on the guideline of Hansen and Perry (1994), counts were indicative of moderate infections in the untreated goats removed from pasture at day 7, but only light infections were found in the other two sets of goats. While small numbers (overall mean count: 6 ± 1 worms) of *Trichostrongylus colubriformis*, *Strongyloides papillosus*, *Nematodirus spathiger* and *Trichuris* spp. were recovered from the goats, these worms were probably present in the animals at the time they were placed on pasture. There were no significant differences between control and COWP-treated groups for the mean total counts for the other nematode species (*P* > 0.08), but differences were found between treatments for *H. contortus*. In the group removed from pasture on day 7 following treatment there was a significant reduction of 71% in the *H. contortus* counts in the treated goats compared with the controls (*P* = 0.004). While the percentage reduction in *H. contortus* burden in the group removed from pasture on day 28 was 37%, this reduction was not significant (*P* = 0.945). The *H. contortus* counts in the COWP-treated goats were higher in the goats removed from pasture on day 56 relative to the untreated goats, but this difference was not significant (*P* = 0.665).

## 4 Discussion

The study investigated the persistence of efficacy of COWP against *H. contortus* in sets of goats grazed on common infective pastures and serially removed from pasture 7, 28 and 56 days post treatment. Copper levels were measured in the organs at slaughter to give an indication of the frequency at which the product might be re-administered. Liver copper values of 25–150 ppm wet weight are considered adequate in goats, while values of 180–250 ppm are considered high (Puls, 1994). As such, only the COWP 7 d (191 ± 19.7 ppm) and COWP 56 d goats (163 ± 20.3 ppm) had values that were between the adequate and high range. The mean kidney copper levels were within the range of 3.0–6.0 ppm wet weight defined as adequate by Puls (1994). Puls (1994) does not specify values for copper levels in muscle tissue for goats, but indicates that values of 1.0–1.3 ppm wet weight are considered adequate in sheep, while values of 1.1–1.6 ppm are considered high. The mean values for the goats in the present study were thus within the adequate range. Copper levels in the faeces in treated animals did not differ significantly from those of untreated goats. Repeat treatments with 4 g COWP should thus be possible without the danger of copper toxicity 84 days (56 days + 28 days) after initial treatment.

The *H. contortus* counts indicate that at most the efficacy of treatment with COWP extended for 28 days post treatment. Five other studies provide indications of the lack of persistence of the anthelmintic effects of COWP beyond 28 days in goats based on worm counts (Burke et al., 2010; Chartier et al., 2000; Martínez-Ortiz-de-Montellano et al., 2007; Soli et al., 2010; Vatta et al., 2009). In sheep, the persistence of the efficacy of COWP seems to be similarly limited to at most 47 days, but results have been variable (Galindo-Barboza et al., 2011; Knox, 2002; Waller et al., 2004). Only one of these studies (Galindo-Barboza et al., 2011) had the evaluation of the persistence of the efficacy of COWP as one of the study's main aims and this study was conducted in sheep. The present study is the first to examine specifically the extended effect of COWP in goats, through worm recovery from groups of animals at set intervals after treatment.

The present study also provides valuable information for the potential integration of the use of COWP in worm control strategies by farmers, as the study was carried out under natural grazing conditions. Although the infectivity of pastures will vary under natural conditions over a period of time, as it did in the present study, with the control goats removed from pasture at day 56 harbouring fewer parasites than those removed at day 7, this type of variation is representative of actual farming conditions. The data presented here support the findings of a recent field study in indigenous goats (Spickett et al., 2012). These authors investigated the use of COWP as a treatment in the mid-summer to prevent the expected peak in FECs and the concomitant contamination of pasture. They found a significant decrease in FECs at 14 days after treatment with 4 g COWP compared with controls and improved PCVs at 14 and 42 days. While their findings were based on FEC and PCV data only, the present study supports these efficacy findings with worm count data in addition to FEC and PCV data. In the present study, FECs were lower and PCVs were higher in COWP-treated goats than controls up to 26 and 47 days post treatment, respectively.

It is widely accepted that *H. contortus* is pathogenic, and therefore potentially surprising that reduction of the parasite burden is not manifest in terms of growth rate, as the administration of COWP had no effect on the live weight of the animals in the present study. The effects on live weight after COWP treatment have been inconsistent between studies, with treated animals gaining more weight than controls in one of the experiments described by Knox (2002) and in one of the treated groups in one of the experiments by Vatta et al. (2009), but no differences being seen between groups in studies by Burke et al. (2004), Martínez-Ortiz-de-Montellano et al. (2007) and Galindo-Barboza et al. (2011). While any beneficial effects of COWP-treatment on live weight would be expected to occur through the elimination of the erosive effects of the parasites, the inconsistency of results suggests that factors such as nutrition, environmental conditions (such as season), frequency of COWP treatment, dosage of COWP, worm burdens at treatment, parasite species and levels of subsequent reinfection play important roles in determining the final effect on productivity.

Anthelmintic resistance was described previously in the *H. contortus* population on the experimental farm from which the goats were purchased for the present experiment. Resistance to oxfendazole, levamisole, morantel and rafoxanide (in sheep grazed on the farm before the goats were introduced; Van Wyk et al., 1989) and to combinations of fenbendazole and levamisole, and trichlorphon and ivermectin (Vatta et al., 2009). Vatta et al. (2009) found that moxidectin was still effective at 0.4 mg/kg. The results of the present investigation, however, indicate resistance to the combination of levamisole and rafoxanide, as well as to moxidectin. Some of the goats in the study had apparently been transferred from another government experimental farm in the same province to the farm in Pietermaritzburg before all the goats were transported to Onderstepoort Veterinary Institute. As such, it is not clear whether the resistant nematodes found in the goats truly represent the population on the Pietermaritzburg farm, and the current status of anthelmintic resistance should be assessed on-farm.

This study has demonstrated the lack of persistence of efficacy of COWP beyond 28 days, but has confirmed its usefulness as an anthelmintic to reduce pasture contamination at times of high nematode transmission. COWP may be used effectively in conjunction with conventional anthelmintics, through the use of the FAMACHA^©^ system (Spickett et al., 2012), or potentially with other alternative strategies for worm control, such as the tannin-containing forage, sericea lespedeza (Burke et al., 2012). On farms where all conventional anthelmintics have failed due to resistance and the novel anthelmintics monepantel and derquantel are not yet available, individual COWP treatments could potentially be administered to anaemic animals based on the FAMACHA^©^ system as described by Burke et al. (2012). Burke and Miller (2006) found dosages as low as 0.5 g effective in lambs and repeated the treatments at 0, 42, 84 and 126 days without risk of copper toxicity. Further work should investigate the use of lower dosages of COWP and repeated administration of COWP in indigenous goats.

## Figures and Tables

**Table 1:** The mean faecal egg counts (FECs) in eggs per gram of faeces (epg) and the corresponding percentages reduction in FEC following anthelmintic treatment of goats purchased from an experimental farm near Pietermaritzburg, South Africa.

Day	Mean FEC in epg (range)	Percentage reduction^a^	*n*
−117	1069 (100–5200)	–	42^b^
−114	7.5 mg/kg levamisole and 7.5 mg/kg rafoxanide (Nem-a-rid^®^, Intervet South Africa)	–	42
−105	317 (0–1500)	40%	42

−105	493 (100–1500)	–	27^b^
−100	0.4 mg/kg moxidectin (Cydectin Injectable^®^, Fort Dodge Animal Health)	–	27
−86	41 (0–500)	85%	27

a Mean percentage reduction where percentage reduction = (FEC_1_ − FEC_2_)/FEC_1_ × 100 where 1 represents the FEC pre treatment and 2 the FEC post treatment (Van Wyk and Van Wijk, 1992). The percentage reduction per individual goat was first calculated before the mean for the group was determined (Cabaret and Berrag, 2004).

b Only data for which FECs were available on each date and for which the pre treatment FECs were greater than zero were included in the calculations.

**Table 2:** Mean copper levels ± standard errors of the mean in parts per million (ppm) on a wet basis in tissues and faeces for groups of goats (*n* = 8) treated with 4 g copper oxide wire particles (COWP; day 0) and removed from *Haemonchus contortus*-seeded pasture on day 7 (7 d), day 28 (28 d) or day 56 (56 d) post treatment and the respective control groups (CONTROL). Goats were maintained in concrete pens and slaughtered 27 or 29 days after removal from pasture. Samples were collected at slaughter.

Group	Liver	*P*^a^	Kidney	*P*^a^	Muscle	*P*^a^	Faeces	*P*^a^
CONTROL 7 d	120 ± 19.7		3.75 ± 0.341		1.00 ± 0.154		16.6 ± 2.47	
COWP 7 d	191 ± 19.7	0.022	3.75 ± 0.341	1.000	1.10 ± 0.154	0.652	15.2 ± 2.47	0.700

CONTROL 28 d	121 ± 9.5		3.75 ± 0.146		0.90 ± 0.113		12.5 ± 1.52	
COWP 28 d	148 ± 9.5	0.060	4.12 ± 0.146	0.090	1.13 ± 0.113	0.182	15.0 ± 1.52	0.265

CONTROL 56 d	150 ± 20.3		3.75 ± 0.222		0.93 ± 0.157		24.5 ± 2.96	
COWP 56 d	163 ± 20.3	0.651	4.00 ± 0.222	0.438	0.85 ± 0.157	0.741	17.9 ± 2.96	0.135

a *P* value for comparison of mean values between groups removed from pasture on the same day.

**Table 3:** Mean *Haemonchus contortus* (*H. c.*) counts ± standard errors of the mean for groups of goats (*n* = 8) treated with 4 g copper oxide wire particles (COWP; day 0) or not treated (CONTROL) and removed from *H. contortus*-seeded pasture on day 7 (7 d), day 28 (28 d) or day 56 (56 d) post treatment. Goats were maintained in concrete pens and slaughtered 27 or 29 days after removal from pasture.

Group	Total *H. c.* count ± SD (L_4_ count ± SD)	PR^a^	*P* value *H. c.* count (*P* value L_4_ count)^b^
CONTROL 7 d	645 ± 152 (76 ± 52)		
COWP 7 d	184 ± 48 (42 ± 23)	71%	0.004 (0.482)

CONTROL 28 d	331 ± 156 (32 ± 10)		
COWP 28 d	207 ± 42 (15 ± 7)	37%	0.945 (0.046)

CONTROL 56 d	225 ± 53 (39 ± 11)		
COWP 56 d	336 ± 89 (19 ± 6)	−49%	0.665 (0.166)

a Percentage reduction in *H. contortus* counts.

b *P* value for comparison of mean total *H. contortus* counts (*P* value for comparison of mean *H. contortus* fourth-stage larval counts) between groups removed from pasture on the same day.

## References

[bib0005] Bang K.S., Familton A.S., Sykes A.R. (1990). Effect of copper oxide wire particle treatment on establishment of major gastrointestinal nematodes in lambs. Res. Vet. Sci..

[bib0010] Barger I.A., Le Jambre L.F., Georgi J.R., Davies H.I. (1985). Regulation of *Haemonchus contortus* populations in sheep exposed to continuous infection. Int. J. Parasitol..

[bib0015] Boyazoglu P.A., Barrett E.L., Young E., Ebedes H. (1972). Liver mineral analysis as indicator of nutritional adequacy. Proceedings of the 2nd World Congress on Animal Feeding.

[bib0020] Burke J.M., Miller J.E. (2006). Evaluation of multiple low doses of copper oxide wire particles compared with levamisole for control of *Haemonchus contortus* in lambs. Vet. Parasitol..

[bib0025] Burke J.M., Miller J.E., Mosjidis J.A., Terrill T.H. (2012). Use of a mixed sericea lespedeza and grass pasture system for control of gastrointestinal nematodes in lambs and kids. Vet. Parasitol..

[bib0030] Burke J.M., Miller J.E., Olcott D.D., Olcott B.M., Terrill T.H. (2004). Effects of copper oxide wire particles dosage and feed supplement level on *Haemonchus contortus* infection in lambs. Vet. Parasitol..

[bib0035] Burke J.M., Soli F., Miller J.E., Terrill T.H., Wildeus S., Shaik S.A., Getz W.R., Vanguru M. (2010). Administration of copper oxide wire particles in a capsule or feed for gastrointestinal nematode control in goats. Vet. Parasitol..

[bib0040] Burke J.M., Terrill T.H., Kallu R.R., Miller J.E., Mosjidis J. (2007). Use of copper oxide wire particles to control gastrointestinal nematodes in goats. J. Anim. Sci..

[bib0045] Cabaret J., Berrag B. (2004). Faecal egg count reduction test for assessing anthelmintic efficacy: average versus individually based estimations. Vet. Parasitol..

[bib0050] Chartier C., Etter E., Hoste H., Pors I., Kock C., Dellac B. (2000). Efficacy of copper oxide needles for the control of nematode parasites in dairy goats. Vet. Res. Commun..

[bib0055] Dobson R.J., Waller P.J., Donald A.D. (1990). Population dynamics of *Trichostrongylus colubriformis* in sheep: the effect of infection rate on the establishment of infective larvae and parasite fecundity. Int. J. Parasitol..

[bib0060] Galindo-Barboza A.J., Torres-Acosta J.F.J., Cámara-Sarmiento R., Sandoval-Castro C.A., Aguilar-Caballero A.J., Ojeda-Robertos N.F., Reyes-Ramírez R., España-España E. (2011). Persistence of the efficacy of copper oxide wire particles against *Haemonchus contortus* in sheep. Vet. Parasitol..

[bib0065] Hansen J., Perry B. (1994). The Epidemiology, Diagnosis and Control of Helminth Parasites of Ruminants. A Handbook.

[bib0070] Knox M.R. (2002). Effectiveness of copper oxide wire particles for *Haemonchus contortus* control in sheep. Aust. Vet. J..

[bib0075] Martínez-Ortiz-de-Montellano C., Vargas-Magaña J.J., Aguilar-Caballero A.J., Sandoval-Castro C.A., Cob-Galera L., May-Martínez M., Miranda-Soberanis R., Hoste H., Cámara Sarmiento R., Torres-Acosta J.F.J. (2007). Combining the effects of supplementary feeding and copper oxide needles for the control of gastrointestinal nematodes in browsing goats. Vet. Parasitol..

[bib0080] Ministry of Agriculture, Fisheries and Food (1986). Manual of Veterinary Parasitological Laboratory Techniques.

[bib0085] Payne R.W., Murray D.A., Harding S.A., Baird D.B., Soutar D.M. (2011). Introduction to GenStat^®^ for Windows™.

[bib0090] Payne R.W., Welham S.J., Harding S.A. (2011). A Guide to REML in GenStat^®^.

[bib0095] Puls R. (1994). Mineral Levels in Animal Health. Diagnostic Data.

[bib0100] Reinecke R.K. (1983). Veterinary Helminthology.

[bib0105] Russell A. (1984). Body condition scoring of sheep. In Practice.

[bib0110] Soli F., Terrill T.H., Shaik S.A., Getz W.R., Miller J.E., Vanguru M., Burke J.M. (2010). Efficacy of copper oxide wire particles against gastrointestinal nematodes in sheep and goats. Vet. Parasitol..

[bib0115] Spickett A., De Villiers J.F., Boomker J., Githiori J.B., Medley G.F., Stenson M.O., Waller P.J., Calitz F.J., Vatta A.F. (2012). Tactical treatment with copper oxide wire particles and symptomatic levamisole treatment using the FAMACHA© system in indigenous goats in South Africa. Vet. Parasitol..

[bib0120] Van Wyk J.A., Van Wijk E.F. (1992). Weerstand van klein strongiele van ‘n perdestoetery in Suid-Afrika teen die bensimidasool wurmmiddels. J. S. Afr. Vet. Assoc..

[bib0125] Van Wyk J.A., Cabaret J., Michael L.M. (2004). Morphological identification of nematode larvae of small ruminants and cattle simplified. Vet. Parasitol..

[bib0130] Van Wyk J.A., Van Schalkwyk P.C., Gerber H.M., Visser E.L., Alves R.M.R., Van Schalkwyk L. (1989). South African field strains of *Haemonchus contortus* resistant to the levamisole/morantel group of anthelmintics. Onderstepoort J. Vet. Res..

[bib0135] Vatta A.F., De Villiers J.F., Gumede S.A., Krecek R.C., Mapeyi N.P., Pearson R.A., Smith M.F., Stenson M.O., Harrison L.J.S. (2007). Benefits of urea-molasses block supplementation and symptomatic and tactical anthelmintic treatments of communally grazed indigenous goats in the Bulwer area, KwaZulu-Natal Province, South Africa. J. S. Afr. Vet. Assoc..

[bib0140] Vatta A.F., Waller P.J., Githiori J.B., Medley G.F. (2009). The potential to control *Haemonchus contortus* in indigenous South African goats with copper oxide wire particles. Vet. Parasitol..

[bib0145] Visser E.L., Van Wyk J.A., Alves R.M.R., Schröder J. (1987). Die identifikasie van die belangrikste nematode. Proceedings of the Worm Resistance Workshop.

[bib0150] Waller P.J., Bernes G., Rudby-Martin L., Ljungström B.-L., Rydzik A. (2004). Evaluation of copper supplementation to control *Haemonchus contortus* infections of sheep in Sweden. Acta Vet. Scand..

[bib0155] Williams C.S.F. (1990). Routine sheep and goat procedures. Vet. Clin. North Am. Food Anim. Pract..

[bib0160] Wood I.B., Amaral N.K., Bairden K., Duncan J.L., Kassai T., Malone J.B., Pankavich J.A., Reinecke R.K., Slocombe O., Taylor S.M., Vercruysse J. (1995). World Association for the Advancement of Veterinary Parasitology (W.A.A.V.P.) second edition of guidelines for evaluating the efficacy of anthelmintics in ruminants (bovine, ovine, caprine). Vet. Parasitol..

[bib0165] Wright W.H., Bozicevich J. (1931). Control of gastrointestinal parasites of sheep by weekly treatments with various anthelmintics. J. Agric. Res..

